# Prediction of postoperative hypokalemia in patients with oral cancer undergoing en bloc cancer resection: a retrospective cohort study

**DOI:** 10.1186/s12903-023-03371-7

**Published:** 2023-09-14

**Authors:** Qilin Bao, Lei Song, Liyuan Ma, Meng Wang, Zhaohuan Hou, Jie Lin, Chunjie Li

**Affiliations:** 1 Nutrition Department, West China Hospital of Stomatology, Sichuan University, State Key Laboratory of Oral Diseases, National Clinical Research Center for Oral Diseases, No. 14, Section Three, Ren Min Nan Road, Chengdu, Sichuan China; 2 Medical Record Room, West China Hospital of Stomatology, Sichuan University, State Key Laboratory of Oral Diseases, National Clinical Research Center for Oral Diseases, No. 14, Section Three, Ren Min Nan Road, Chengdu, Sichuan China; 3 Department of Head and Neck Oncology, West China Hospital of Stomatology, Sichuan University, State Key Laboratory of Oral Diseases, National Clinical Research Center for Oral Diseases, No. 14, Section Three, Ren Min Nan Road, Chengdu, Sichuan China; 4 Department of Oral Anesthesia, West China Hospital of Stomatology, Sichuan University, State Key Laboratory of Oral Diseases, National Clinical Research Center for Oral Diseases, No. 14, Section Three, Ren Min Nan Road, Chengdu, Sichuan China

**Keywords:** Postoperative hypokalemia, Platelet-to-lymphocyte ratio, Nomogram, Oral cancer, Postoperative complication

## Abstract

**Background:**

The factors associated with postoperative hypokalemia in patients with oral cancer remain unclear. We determined the preoperative factors associated with postoperative hypokalemia in patients with oral cancer following en bloc cancer resection and established a nomogram for postoperative hypokalemia prediction.

**Methods:**

Data from 381 patients with oral cancer who underwent en bloc cancer resection were retrospectively analyzed. Univariate and multivariate analyses were performed to identify the risk factors for postoperative hypokalemia. We used receiver operating characteristic (ROC) curves to quantify the factors’ effectiveness. A nomogram was created to show each predictor’s relative weight and the likelihood of postoperative hypokalemia development. The multinomial regression model’s effectiveness was also evaluated.

**Results:**

Preoperative factors, including sex, preoperative serum potassium level, and preoperative platelet-to-lymphocyte ratio (PLR), were significantly associated with postoperative hypokalemia. Based on the ROC curve, the preoperative serum potassium and PLR cut-off levels were 3.98 mmol/L and 117, respectively. Further multivariate analysis indicated that female sex, preoperative serum potassium level < 3.98 mmol/L, and preoperative PLR ≥ 117 were independently associated with postoperative hypokalemia. We constructed a predictive nomogram with all these factors for the risk of postoperative hypokalemia with good discrimination and internal validation.

**Conclusions:**

The predictive nomogram for postoperative hypokalemia risk constructed with these factors had good discrimination and internal validation. The developed nomogram will add value to these independent risk factors that can be identified at admission in order to predict postoperative hypokalemia.

## Background

Oral cancer is the main category of head and neck cancers and the sixth most common malignancy worldwide; it is distinguished by a high locoregional recurrence rate and low long-term survival rates [1]. Studies have shown that surgical management is one of the primary treatment modalities necessary for achieving optimal survival outcomes in patients with oral cancer [2, 3]. The principal surgical treatment for advanced oral cancer is en bloc cancer resection; however, thorough resection leaves a significant defect that has a substantial impact on the intricate structures and functions of the oral and maxillofacial areas [4, 5]. Moreover, extensive resection might cause malformation, disfigurement, and dysfunction [6]. For these patients, gastric tubes are used to help with nutritional treatment and to maintain a clean oral cavity environment. Because of feeding through gastric tubes, the change in nutrition supply always induces different fluid levels and electrolyte imbalances. In our clinical practice, we have noticed that patients with oral cancer who undergo en bloc cancer resection often develop hypokalemia after surgery, despite having a normal preoperative serum potassium level (3.5–5.3 mmol/L).

Hypokalemia is a common electrolyte disorder with a serum potassium of less than 3.5 mmol/L, caused by inadequate potassium intake or high potassium loss [7]. Mild hypokalemia with a serum potassium concentration of 3.0 mmol/L to 3.5 mmol/L may have no obvious clinical symptoms, however, it has been shown that mild hypokalemia increases the risk of stroke and mortality in the general population [8]. As serum potassium level falls further, moderate and severe hypokalemia may cause complications such as muscle weakness, paralytic ileus, metabolic acidosis, rhabdomyolysis, arrhythmia, and even death [7, 9–11]. Perioperative hypokalemia increases the potential for delayed recovery of gastrointestinal function and severe cardiovascular events in oral cancer patients [12]. Persistent hypokalemia is an independent predictor of mortality and unfavorable cardiovascular events within 30 days of noncardiac surgery [13]. Thus, diligent monitoring and correction of hypokalemia may play a crucial role in improving patients' prognoses. However, research on the association between potential risk factors and postoperative hypokalemia in patients with oral cancer is lacking.

Thus, the primary objective of this study was to determine whether preoperative factors associated with potassium intake and loss were related to postoperative hypokalemia in patients with oral cancer who underwent en bloc cancer resection and to provide a nomogram for postoperative hypokalemia prediction.

## Methods

### Patient population and study design

This study used a retrospective cohort design. Medical records were retrieved for all patients with an International Classification of Diseases-10 oral cancer diagnosis from January 2020 to June 2021 at our center. Double entry is used for data extraction. The Ethics Committee of West China Hospital of Stomatology, Sichuan University (protocol number WCHSIRB-CT-2021–365; August 12, 2021) approved this study, which was conducted in accordance with the Declaration of Helsinki. Due to the study’s retrospective nature and use of anonymized data, informed consent was not required.

Patients were considered in this study if they satisfied the following criteria: (1) patients with oral cancer who underwent en bloc cancer resection and used gastric tubes after surgery in the Department of Head and Neck Oncology, West China Hospital of Stomatology, Sichuan University; (2) patients with no history of gastrointestinal bleeding or blood transfusion after surgery; and (3) patients whose electrolytes were regularly assessed after surgery. We identified a total of 393 patients with oral cancer who underwent en bloc cancer resection at our center between January 2020 and June 2021. One patient was excluded owing to a history of blood transfusion after surgery and 11 patients were excluded owing to non-regular assessment of electrolytes post-surgery. Thus, a total of 381 patients were enrolled in this study with complete data.

Patients were further allocated to a model-development set (January 2020 to December 2020) and a validation set (January 2021 to June 2021) based on admission dates.

### Study variables

Each patient’s chart was reviewed after the appropriate medical documents were obtained. Demographic and clinical data were collected, including age, sex, height, weight, history of systemic diseases such as hypertension and diabetes, cancer site, oral cancer stage, and surgical plan. All preoperative consultation and clinic notes, laboratory data, and operative reports were reviewed. Laboratory data included preoperative serum potassium; serum albumin (ALB); blood platelet levels; and lymphocyte, monocyte, and neutrophil counts. The operative variables included operation time, along with intraoperative infusion (crystalloid fluids and colloid fluids), urinary, and bleeding volumes with the follow equation: bleeding volume = weight of bloody gauze—weight of dry gauze + blood volume in suction bottle (ml). All tumors were staged according to the tumor-node-metastasis classification of the American Joint Committee on Cancer (2017).

The primary outcome variable was postoperative hypokalemia, based on the first postoperative measurement of the serum potassium level, conducted within 48 h after surgery. Hypokalemia was defined as a serum potassium level of < 3.5 mmol/L, it may be classified as mild (serum potassium is between 3.0 mmol/L and 3.5 mmol/L), moderate (serum potassium is between 2.5 mmol/L and 3.0 mmol/L) or severe (serum potassium is below 2.5 mmol) [7, 11]. The baseline serum potassium level was defined as the potassium level in the patient’s serum at the time of admission.

### Assessment of nutrition status and systemic inflammatory response parameters

Patients’ weight and height were used to calculate the body mass index (BMI) with the following equation: BMI = weight/height^2^ (kg/m^2^). Serum ALB levels and total lymphocyte counts measured before surgery were used to calculate the prognostic nutritional index (PNI) using the following equation: PNI = serum ALB (g/L) + 5 × total lymphocyte count (10^9^/L) [14]. We determined the platelet-to-lymphocyte ratio (PLR) by dividing the absolute platelet count by the absolute lymphocyte count, the lymphocyte-to-monocyte ratio (LMR) by dividing the absolute lymphocyte count by the absolute monocyte count, and the neutrophil-to-lymphocyte ratio (NLR) by dividing the absolute neutrophil count by the absolute lymphocyte count [15].

### Sample size

The effective sample size in prediction research was determined by the number of outcome events [16]. Hyperkalemia prevalence has been reported at approximately 20% in hospitalized patients in previous literature [17]. Based on the model's sample size requirement [18, 19], the size of the sample is calculated in R software using the package “pmsampsize”. A multivariate regression model requiring 10 or fewer predictors would require 263 or more patients. It should be possible to generate reliable estimates with the number of patients we included.

### Statistical analyses

Continuous variables were expressed as mean and standard deviations or medians with range. The results for categorical variables are described using frequencies and percentages. The chi-squared test or Fisher’s exact test was used to compare the discrete variables. A Student’s t-test or Mann-Whiney U test was employed to compare the cohort's quantitative data. We performed an explanatory analysis using the univariate/multivariate Poisson and logistic regression approaches. Multicollinearity can be detected using the variance inflation factor (VIF). Variables related to a significant change (*P* < 0.05) at univariate analysis were further analyzed using multivariate Poisson’s or logistic regression. A multivariate logistic regression was used for risk score modeling via variable selection. Data were reported with relative risks (RRs) and 95% confidence intervals. Receiver operating characteristic (ROC) curves were generated to evaluate the sensitivity and specificity of the preoperative serum potassium level and preoperative PLR for predicting postoperative hypokalemia, and Youden’s index was estimated to determine the optimal cut-off value for the preoperative serum potassium level and PLR.

A nomogram was developed to identify patients at risk of developing postoperative hypokalemia. It provided a graphical representation of the effect, which can be used to calculate the risk of postoperative hypokalemia development for an individual patient based on the points associated with each risk factor. The model-development set and the validation set were both used to internally and externally validate the nomogram. The external validation in this study was temporal validation, depending on the source of the cohort data. We used a calibration technique and the area under the ROC curve (AUC) to carry out the internal validation. The AUC calculation served as external validation. Statistical differences between the different AUCs were investigated using the DeLong method. A calibration plot illustrates the association between the actual and predicted probabilities. Only complete data is analyzed. All analyses were performed using SPSS version 26.0 (SPSS Inc., Chicago, IL, USA) and R version 4.1.0 (The R Foundation for Statistical Computing, Vienna, Austria), with the statistical significance threshold of *P* < 0.05.

## Results

### Characteristics of the included patients

The study comprised a total of 381 patients who met the eligibility criteria. Moreover, 253 and 128 patients were classified into the model-development and validation sets, respectively, according to the admission date. The baseline characteristics of the patients are summarized in Table 1.

**Table 1:** Baseline characteristics of the model-development and validation sets

**Variables**	**Group**	**Model-development set ** **N (253)**	**Validation set** **N(128)**	***P* value**
Age, median(range), year		61(16–84)	60.5(31–79)	0.599
Sex	Male	160(63.2%)	87(68.0%)	0.367
	Female	93(36.8%)	41(32.0%)	
BMI (kg/m^2^)		23.06 ± 3.34	23.73 ± 3.11	0.060
Diabetes mellitus	Yes	25(9.9%)	17(13.3%)	0.386
	No	228(90.1%)	111(86.7%)	
Hypertension	Yes	45(17.8%)	34(26.6%)	0.061
	No	208(82.2%)	94(73.4%)	
Preoperative serum potassium, median(range), mmol/L		4.01(2.98–5.20)	3.97(3.16–4.80)	0.609
Preoperative ALB, median(range)		42.15(31.6–50.5)	42.6(36.7–49.1)	0.102
Preoperative PNI, median(range)		49.90(35.75–66.75)	50.43(14.59–59.45)	0.304
Preoperative LMR, median(range)		3.54(0.82–10.91)	3.71(0.39–8.62)	0.170
Preoperative NLR, median(range)		2.25(0.47–10.12)	2.29(0.89–24.68)	0.242
Preoperative PLR, median(range)		123.7(43.5–614.3)	129.0(46.5–733.3)	0.170
Intraoperative infusion volume, median(range), ml		2975(500–5300)	2575(800–5400)	0.167
Intraoperative crystalloid solution, median(range), ml		2025(200–5300)	2000(700–4100)	0.514
Intraoperative colloidal solution, median(range), ml		1000(0–2000)	775(0–2000)	0.117
Intraoperative potassium supplementation, median(range), g		0.6(0.06–1.59)	0.6(0.21–1.23)	0.620
Bleeding volume, median(range), ml		400(30–1100)	400(50–4000)	0.331
Intraoperative urinary volume, median(range), ml		650(0–3250)	600(0–2500)	0.715
Operation time, median(range), min		325(49–710)	305(85–800)	0.561
ASA score	I	17(6.7%)	16(12.5%)	0.056
	II	217(85.8%)	96(75.0%)	
	III	19(7.5%)	16(12.6%)	
	IV	0(0.0%)	0(0.0%)	
	V	0(0.0%)	0(0.0%)	
TNM stage	I	23(9.1%)	11(8.6%)	0.467
	II	35(13.8%)	24(18.8%)	
	III	42(16.6%)	25(19.5%)	
	IV	153(60.5%)	68(53.1%)	
Tumor site	Tongue	90(35.6%)	59(46.1%)	0.243
	Cheek	78(30.8%)	29(22.7%)	
	Gingiva	36(14.2%)	12(9.4%)	
	Floor of mouth	37(14.6%)	19(14.8)	
	Oropharynx	8(3.2%)	6(4.7%)	
	Palate	1(0.4%)	0(0.0%)	
	Mandible	3(1.2%)	3(2.3%)	

*Abbreviations*: *BMI* Body mass index, *ALB* Albumin, *PNI* Prognostic nutrition index, *LMR* Lymphocyte-monocyte ratio, *NLR* neutrophil-to-lymphocyte ratio, *PLR* Platelet-to-lymphocyte ratio, *ASA score* American Society of Anesthesiologists score, *TNM* Tumor node metastasis

### Univariate analyses for factors related to postoperative hypokalemia in the model-development set

Of all the included patients in the model-development set, 85 developed hypokalemia postoperatively. The association of demographic data, nutritional evaluation indicators, laboratory data, inflammatory factors, and intraoperative indicators with postoperative hypokalemia was investigated. Univariate analyses indicated that preoperative serum potassium level (*P* = 0.001), preoperative PLR (*P* = 0.002), and sex (*P* < 0.001) were significantly associated with postoperative hypokalemia (Table 2).

**Table 2:** Descriptive characteristics and association with postoperative hypokalemia in model-development set

**Variable**	**Postoperative hypokalemia (*n* = 85)**	**Normal (*n* = 168)**	***P* value**
Age, median(range),years	61(26–83)	60(16–84)	0.435
BMI(kg/m^2^), mean ± SD	23.23 ± 3.16	22.97 ± 3.42	0.567
Preoperative serum potassium level, median(range), mmol/L	3.91(2.98–4.61)	4.06(3.03–5.20)	0.001
Preoperative ALB, mean ± SD	42. 31 ± 2.88	42.05 ± 3.05	0.514
Preoperative PNI, mean ± SD	50.09 ± 4.52	50.37 ± 4.50	0.506
Preoperative LMR, median(range)	3.69(1.24–6.90)	3.50(0.82–10.91)	0.431
Preoperative NLR, median(range)	2.34(0.58–6.13)	2.22(0.27–10.12)	0.281
Preoperative PLR, median(range)	134.6(50.0–287.1)	112.9(43.5–614.3)	0.002
Intraoperative infusion volume (ml), mean ± SD	2941 ± 845	2921 ± 1063	0.737
Intraoperative crystalloid solution, median(range), ml	2100(700–3500)	2000(200–5300)	0.750
Intraoperative colloidal solution, median(range), ml	900(0–2000)	1000(0–2000)	0.742
Intraoperative potassium supplementation, median(range), g	0.63(0.21–1.05)	0.61(0.60–1.59)	0.919
Bleeding volume, median(range), ml	400(30–800)	400(50–1100)	0.527
Intraoperative urinary volume, median (range), ml	700(0–3250)	650(0–2250)	0.434
Operation time, median(range), min	340(90–710)	315(49–680)	0.385
Sex			0.000
Male	39(45.9%)	121(72.0%)	
Female	46(54.1%)	47(28.0%)	
Diabetes mellitus			0.533
Yes	7(8.2%)	18(10.7%)	
No	78(91.8%)	150(89.3%)	
Hypertension			0.500
Yes	13(15.3%)	33(19.6%)	
No	72(84.7%)	135(80.4%)	
Tumor site			0.435
Tongue	34(40.0%)	56(33.3%)	
Buccal	30(35.3%)	48(28.6%)	
Gingiva	11(12.9%)	25(14.9%)	
Floor of mouth	8(9.4%)	29(17.3%)	
Oropharynx	2(2.4%)	6(3.6%)	
Palate	0(0.0%)	1(0.6%)	
Mandible	0(0.0%)	3(1.8%)	
TNM stage			0.566
I	10(11.8%)	13(7.7%)	
II	9(10.6%)	26(15.5%)	
III	15(17.6%)	27(16.1%)	
IV	51(60.0%)	102(60.7%)	
ASA score			0.700
I	6(7.1%)	11(6.5%)	
II	71(83.5%)	146(86.9%)	
III	8(9.4%)	11(6.5%)	
IV	0(0.0%)	0(0.0%)	
V	0(0.0%)	0(0.0%)	

*Abbreviations*: *BMI* Body mass index, *ALB* Albumin, *PNI* Prognostic nutrition index, *LMR* Lymphocyte-monocyte ratio, *NLR* Neutrophil-to-lymphocyte ratio, *PLR* Platelet-to-lymphocyte ratio, *ASA score* American Society of Anesthesiologists score, *TNM* Tumor node metastasis

### Univariate analyses for factors related to postoperative hypokalemia in the model-development set excluding patients with preoperative hypokalemia

In the model-development set, eight patients had hypokalemia at admission. After excluding patients who received preoperative potassium supplementation, we compared the demographic and clinical characteristics of patients with normal preoperative potassium levels in the model-development set. Univariate analyses indicated that preoperative serum potassium level (*P* < 0.001), preoperative PLR (*P* = 0.001), and sex (*P* < 0.001) were significantly associated with postoperative hypokalemia (Table 3).

**Table 3:** Descriptive characteristics and association with postoperative hypokalemia in model-development set without the patients with preoperative hypokalemia

**Variable**	**Postoperative hypokalemia (*n* = 83)**	**Normal (*n* = 162)**	***P* value**
Age, median(range), years	59(16–84)	61(26–83)	0.389
BMI (kg/m^2^), mean ± SD	23.23 ± 3.20	22.92 ± 3.43	0.543
Preoperative serum potassium level, median(range), mmol/L	3.92(2.98–4.61)	4.07(3.03–5.2)	0.000
Preoperative ALB, mean ± SD	42.30 ± 2.91	42.09 ± 3.00	0.305
Preoperative PNI, mean ± SD	50.01 ± 4.47	50.46 ± 4.52	0.618
Preoperative LMR, median(range)	3.68(1.24–6.9)	3.54(0.82–10.91)	0.633
Preoperative NLR, median(range)	2.31(0.58–6.13)	2.19(0.47–10.12)	0.271
Preoperative PLR, median(range)	134.6(50.0–287.1)	112.9(43.5–614.3)	0.001
Intraoperative infusion volume, mean ± SD, ml	2931.08 ± 853.03	2919.94 ± 1054.45	0.722
Intraoperative crystalloid solution, median(range), ml	2100(700–3500)	2000(200–5300)	0.716
Intraoperative colloidal solution, median(range), ml	900(0–2000)	1000(0–2000)	0.926
Intraoperative potassium supplementation, median(range), g	0.63(0.21–1.05)	0.61(0.06–1.59)	0.712
Bleeding volume, median(range), ml	400(30–800)	400(50–1100)	0.411
Intraoperative urinary volume, median(range), ml	700(0–3250)	650(0–2250)	0.524
Operation time, median(range), min	340(90–710)	315(49–480)	0.449
Sex			0.000
Male	39(47%)	117(72%)	
Female	44(53%)	45(27.8)	
Diabetes mellitus			0.657
Yes	7(8.4%)	18(11.1%)	
No	76(91.6%)	144(88.9%)	
Hypertension			0.380
Yes	12(14.5%)	32(19.8%)	
No	71(85.5%)	130(80.2%)	
Tumor site			0.444
Tongue	34(41%)	54(33.3%)	
Buccal	29(34.9%)	47(29.0%)	
Gingiva	10(12.0%)	24(14.8%)	
Floor of mouth	8(9.6%)	28(17.3%)	
Oropharynx	2(2.4%)	6(3.7%)	
Palate	0(0.0%)	1(0.6%)	
Mandible	0(0.0%)	2(0.6%)	
TNM stage			0.623
I	9(10.8%)	13(8.0%)	
II	9(10.8%)	26(16.0%)	
III	15(18.1%)	25(15.4%)	
IV	50(60.2%)	98(60.5%)	
ASA score			0.682
I	6(7.2%)	10(6.2%)	
II	69(83.1%)	141(87.0%)	
III	8(9.8%)	11(6.8%)	
IV	0(0.0%)	0(0.0%)	
V	0(0.0%)	0(0.0%)	

*Abbreviations*: *BMI* Body mass index, *ALB* Albumin, *PNI* Prognostic nutrition index, *LMR* Lymphocyte-monocyte ratio, *NLR* Neutrophil-to-lymphocyte ratio, *PLR* Platelet-to-lymphocyte ratio, *ASA score* American Society of Anesthesiologists score, *TNM* Tumor node metastasis

### ROC curve analysis in the model-development set

**Fig. 1:**
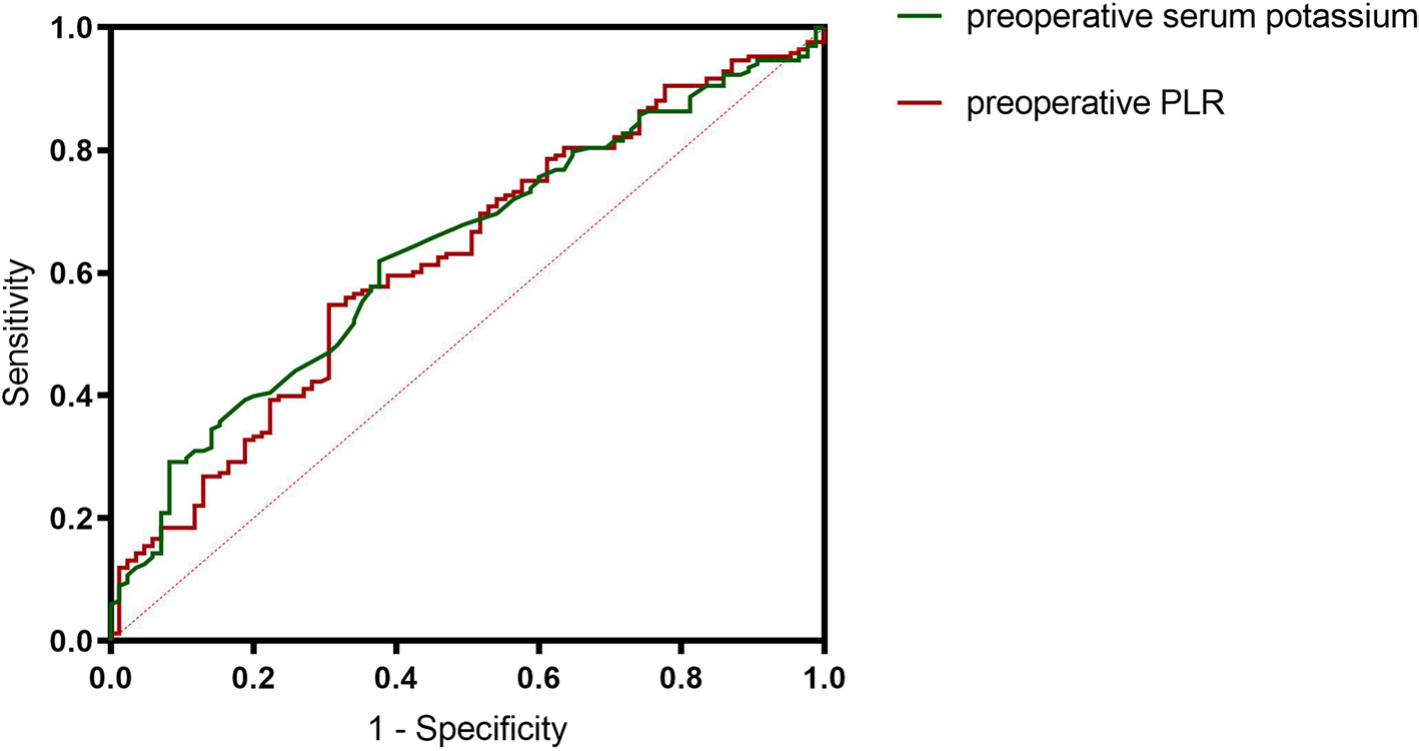
ROC curve for preoperative serum potassium levels and preoperative PLR as risk predictors of postoperative hypokalemia in patients who underwent en-bloc oral cancer resection. Abbreviation: PLR: platelet-to lymphocyte ratio

ROC analyses were performed in the present study to evaluate the utility of the preoperative serum potassium level and preoperative PLR in discriminating postoperative hypokalemia. The optimal cut-off value for preoperative serum potassium was 3.98 mmol/L, with an AUC of 0.63, at 62.4% sensitivity and 61.9% specificity. The optimal cut-off value for preoperative PLR was 117.00, with an AUC of 0.62, at 69.4% sensitivity and 52% specificity (Fig. 1).

### Multivariate analyses and nomogram of factors related to postoperative hypokalemia in the model-development set

**Fig. 2:**
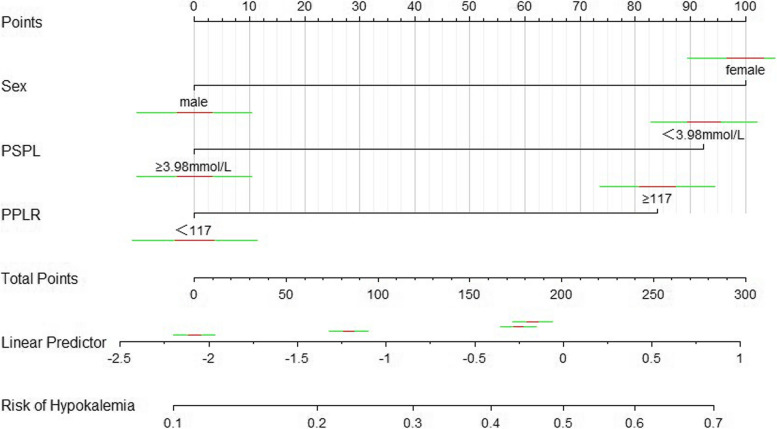
Nomogram to predict the risk of postoperative hypokalemia: to estimate the probability of postoperative hypokalemia of patients with oral cancer who underwent en-bloc resection, the “Total Point” is calculated by summing the respective “Points” values corresponding to each variable. Using this “Total Point”, patients’ probability of postoperative hypokalemia can be predicted according to the scale shown in row 7. Abbreviation: PSPL: preoperative serum potassium level (mmol/L); PPLR: preoperative platelet-to-lymphocyte ratio

The variables included in this analysis had a VIF of less than 10, indicating that there was no multicollinearity. The multivariate logistic regression analyses used all variables that reached statistical significance in the univariate analysis. Multivariate analyses revealed that female sex (*P* = 0.006, relative risk [RR] = 1.81, 95% CI = 1.18–2.79), preoperative serum potassium level < 3.98 mmol/L (*P* = 0.01, relative risk [RR] = 1.76, 95% CI = 1.13–2.74), and preoperative PLR ≥ 117 (*P* = 0.024, relative risk [RR] = 1.71, 95% CI = 1.07–2.74) were independently associated with postoperative hypokalemia in patients with oral cancer who underwent en bloc oral cancer resection. RR with 95%CI for the passion regression model are presented in Table 4. A nomogram incorporating the risk factors was established based on the multivariate analysis results to predict the risk of postoperative hypokalemia (Fig. 2).

**Table 4:** Results of multivariate analysis

**Predictor**	**RR (95%CI)**	***P* value**
Sex (female)	1.81(1.18 to 2.79)	0.006
Preoperative serum potassium level < 3.98 mmol/L	1.76(1.13 to 2.74)	0.010
Pre-operative PLR ≥ 117.00	1.71(1.07 to 2.74)	0.024

### Internal and external validation of the predictive accuracy of the nomogram

**Fig. 3:**
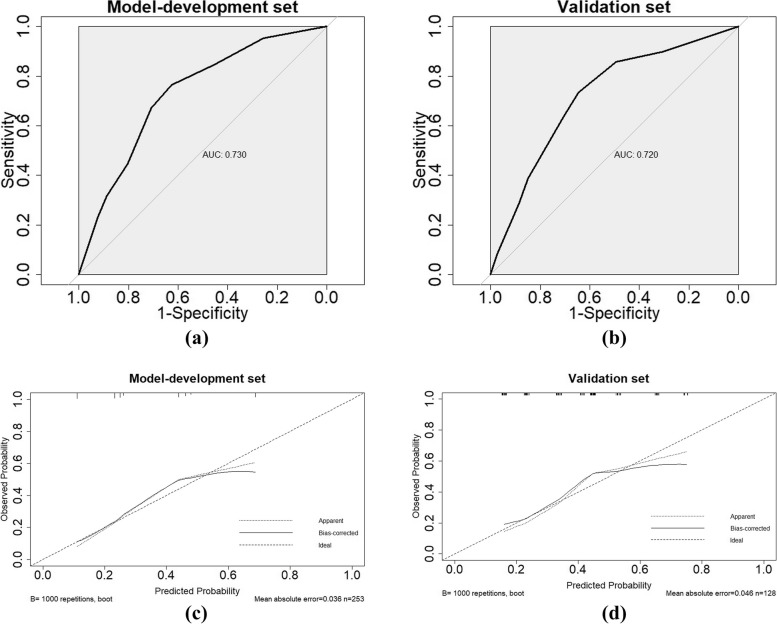
Receiver operating characteristic (ROC) curve and calibration plot for model-development and validation sets of the present nomogram. **a** ROC curve for the model-development set of 253 patients. **b** ROC curve for validation set of 128 patients. **c** Calibration plot for the model-development set. **d** Calibration plot for the validation set. The x-axis represents the nomogram-predicted probability and the y-axis represents the observed rate of postoperative hypokalemia. A perfect prediction would correspond to the 45° dashed line

The model-development set and validation set were applied to the nomogram for internal and external validation, respectively. The AUCs corresponding to the nomogram’s accuracy were 0.730 (95% CI = 0.666–0.793) and 0.720 (95% CI = 0.630–0.810) in the model-development and validation sets, respectively (Fig. 3a, b). There was no statistically significant difference between the two AUCs (*P* = 0.860). These results indicated that there was good agreement between the anticipated and observed probabilities of postoperative hypokalemia, and the nomogram’s goodness of fit was favorable. The calibration plot for the probability of postoperative hypokalemia showed better agreement between the predicted probabilities, indicating the good predictive power of the nomogram when applied to an independent validation data set (Fig. 3c, d).

## Discussion

This study included all patients with oral cancer who underwent en bloc resection and whose serum potassium level 48 h after surgery met the hypokalemia criteria. The model-development set included 253 patients; 85 of these patients had mild hypokalemia (serum potassium = 3.0–3.5 mmol/L) and four had moderate hypokalemia (serum potassium = 2.5–3.0 mmol/L) [7, 11, 20]. Hypokalemia results from insufficient intake, abnormal losses, and transcellular shifts in serum potassium [21, 22]. As no previous reports exist on risk factors related to postoperative hypokalemia in patients with oral cancer who undergo en bloc resection, we examined preoperative factors related to postoperative hypokalemia occurrence in this study. BMI, ALB, and PNI are commonly used to assess nutritional status [23, 24], which is associated with potassium intake [25]. Inflammatory factors are related not only to the nutritional status but also to the prognosis of patients with tumors [26]. The amount of intraoperative fluid replacement affects the patient’s blood volume and acid–base balance. Given that the kidney plays an important role in potassium excretion, intraoperative urine volume was considered an indicator to determine the amount of potassium loss during the surgery [27].

En bloc cancer resection for patients with oral cancer is performed under general anesthesia, and patients usually have an 8-h overnight fast before surgery. After surgery, patients are generally fed through a gastric tube to prevent intraoral surgical wound infection. The time between the operation and the beginning of gastric tube feeding is approximately 10 h. During the perioperative period, the patient’s nutritional intake and amount of food change dramatically, and the amount is usually reduced, which might cause potassium deficiency and starvation. Moreover, glucose levels decline with starvation. Consequently, non-carbohydrate sources (muscle proteins) are metabolized into glucose, and fatty acid oxidation can produce ketone bodies through the Krebs cycle [28]. Under these conditions, significant potassium depletion occurs [29]. The hypokalemia experienced by patients with oral cancer who undergo en bloc cancer resection may result from inadequate oral intake and a shift of potassium from the extracellular fluid to the intracellular fluid. To the best of our knowledge, hypokalemia is one of the most common electrolyte disturbances that contribute to increases in postoperative morbidity, hospital stays, and healthcare burdens, and this condition occurs in approximately 20% of hospitalized patients [17]. In the model-development set, the proportion of patients with postoperative hypokalemia was 33.60% which is higher than that reported in the literature, indicating that postoperative hypokalemia in patients with oral cancer is a complication worthy of clinicians’ attention.

In general, guidelines suggest that patients should be provided with potassium supplements when their serum potassium level is < 3.5 mmol/L [30]. However, patients with oral cancer who undergo en bloc cancer resection often develop hypokalemia after surgery, although their preoperative serum potassium level is within the normal range, highlighting that the optimal potassium level may differ from the current normal range definition. The present study identified and validated the preoperative serum potassium level that is predictive of postoperative hypokalemia in patients with oral cancer treated with en bloc cancer resection. A lower preoperative serum potassium level might be associated with a higher occurrence of postoperative hypokalemia. The ROC curve analysis revealed that a preoperative serum potassium cut-off level of 3.98 mmol/L corresponded to postoperative hypokalemia development. Patients with preoperative serum potassium levels < 3.98 mmol/L were 1.76 times more likely to develop postoperative hypokalemia than patients with preoperative serum potassium levels ≥ 3.98 mmol/L. Previous studies demonstrated that serum potassium levels beyond the range of 4.1–4.7 mmol/L were associated with increased mortality risk. A low normal potassium concentration might be a marker for an ongoing decrease in potassium level [31]. Most researchers favor having a serum potassium level in the 4.5–5.0 mmol/L range. The most efficient and safest intervention for preventing potassium deficiency, which may subsequently impair the function and contractility of myocardial and skeletal muscles, is effective potassium management with appropriately targeted serum potassium concentrations (with eventual depletion of body stores) [32]. Increased potassium consumption should be taken into account when serum potassium levels are between 3.5 and 4.0 mmol/L, according to a previous study [33]. Additionally, Krodager et al. [24] suggested that potassium supplementation in patients with potassium concentrations ≤ 3.7 mmol/L could be of clinical importance. Our results are generally in line with the findings of these previous studies and emphasize that serum potassium values below 3.98 mmol/L are alarming. Our data highlight the significance of promptly treating aberrant serum potassium levels in patients with oral cancer undergoing en bloc cancer excision.

Reduced food intake and various degrees of acute or chronic inflammation lead to altered body function and diminished biological function [26, 34]. Aside from changes in nutrition supply in patients with oral cancer, cancer-related systemic inflammation is essential in all stages of tumor formation, including proliferation, angiogenesis, and metastasis [35]. The PLR is a significant predictive indicator in patients with malignancies, including oral cancer [36, 37]. Low antitumor capacity may be indicated by a high PLR, which is the result of a low lymphocyte count and a high platelet count. This could indicate a poor prognosis [38, 39]. The preoperative PLR cut-off value of 117 was consistent with the predicted value in our study. Patients with higher PLRs were more likely to develop postoperative hypokalemia. The reason for the association between a high preoperative PLR and postoperative hypokalemia development is unclear. Several possible explanations exist. First, the platelet threshold concentration correlates with the serum potassium level [40]. Second, the abundance of Na–K-adenosine triphosphatase and potassium channels in platelets may correlate with the serum potassium level [41]. Given that cancer is mostly a disease of older individuals, the PLR as a significant risk factor (but not the LMR or NLR) is likely due to aging-related immunosurveillance for cancer failing. In fact, 58% of our patients were over 60 years of age, which is consistent with other published series [42, 43]. That said, the average age of the patients in our study was less than 60 years. In addition to clinical parameters, we found that women with oral cancer were more likely to develop hypokalemia after en bloc oral cancer resection than men. Other researchers have reported similar results [44–46]. However, the reason why women develop hypokalemia more often than men is unclear, and no plausible hypotheses exist in the current literature.

For postoperative hypokalemia prediction, we developed a nomogram to visualize the results of the multivariate logistic analyses. Each horizontal line in the nomogram depicts how the predictors have affected the various categories relative to the reference category. Higher scores are represented by longer lines, which also show a bigger impact of the predictor coefficient in that particular category. Disease-specific scores, relating to various patient characteristics, can be “read” in the nomogram for each patient [47, 48]. Our model includes characteristics that are all typically available prior to surgery, which is likely to improve the model's clinical utility across a variety of situations, regardless of infrastructure. Our model demonstrated good discrimination, yielding an AUC of 0.734. From our nomogram, clinicians can predict the likelihood of hypokalemia development in patients with oral cancer after combined radical surgery according to the potassium level at the time of admission and other demographic and clinical characteristics. The nomogram shown herein is preliminary and will need to be independently prospectively validated. Although the current version lacks all necessary variables, we believe it is preferable to no tool and may be applicable to current clinical practice and research.

This study had some limitations. First, insulin use in patients with diabetes and thiazide use in patients with hypertension are considered independent risk factors for hypokalemia [31, 49, 50]. However, the incidence of postoperative hypokalemia in patients with diabetes or hypertension was not comparable in this study, which may be attributed to the limited number of included patients. Second, the focus of our study was to examine the association between preoperative serum potassium levels and outcomes. We lacked data on serial measures or discharge potassium levels; therefore, we could only analyze the influence of entrance potassium levels in the current investigation. Third, because of the limited number of patients and selection from a single center, the predictive value of the independent risk factors in the present study will need to be confirmed in a larger, multicenter cohort study. Notably, the constructed model predicts postoperative hypokalemia in patients with oral cancer. The clinical follow-up of patients with oral cancer undergoing en bloc resection was continued. We will be able to validate our current model clinically with the help of the gathered follow-up data.

## Conclusions

Unnecessary examinations and dangerous hypokalemia-related consequences can be avoided with prompt identification and effective hypokalemia treatment. Therefore, potassium monitoring should be recommended early for female patients, for patients with an admission serum potassium level < 3.98 mmol/L, and for those with a preoperative PLR ≥ 117. We constructed a nomogram to predict the postoperative hypokalemia risk. The model will add value to these independent risk factors that can be identified at admission in order to predict postoperative hypokalemia. Preoperative serum potassium screening and repletion should be considered for patients with oral cancer scheduled for en bloc resection of cancerous tissues. Most importantly, regardless of advancements in technology or treatment methods, the nomogram is meant to remain flexible and adaptable for future patients. It may be constantly updated using a larger sample size, producing a more accurate forecasting tool.

## Data Availability

The data that support the findings of this study are available from the corresponding author upon reasonable request.

## Abbreviations

ALB: Albumin
ASA score: American Society of Anesthesiologists score
BMI: Body mass index
CIs: Confidence intervals
LMR: Lymphocyte-monocyte ratio
NLR: Neutrophil-to-lymphocyte ratio
ORs: Odds ratios
PLR: Platelet-to-lymphocyte ratio
PNI: Prognostic nutrition index
PPLR: Preoperative platelet-to-lymphocyte ratio
PSPL: Preoperative serum potassium level
ROC: Receiver operating characteristic
TNM: Tumor node metastasis
VIF: Variance inflation factor
